# Investigation of Klotho G395A and C1818T Polymorphisms and Their Association with Serum Glucose Level and Risk of Type 2 Diabetes Mellitus

**DOI:** 10.3390/genes13091532

**Published:** 2022-08-26

**Authors:** Muhammad Sadiq Aziz, Aziz-ul-Hasan Aamir, Ajab Khan, Zahid Khan, Syed Qaiser Shah, Sher Zaman Safi, Kalaivani Batumalaie, Hussah M. Alobaid, Abid Ali, Muhammad Imran

**Affiliations:** 1 Biochemistry Section, Institute of Chemical Sciences, University of Peshawar, Peshawar 25120, Pakistan; 2 Department of Diabetes and Endocrinology, Hayatabad Medical Complex, Peshawar 25120, Pakistan; 3 Faculty of Medicine, Bioscience and Nursing, MAHSA University, Jenjarom 42610, Malaysia; 4 Interdisciplinary Research Centre in Biomedical Materials (IRCBM), COMSATS University Islamabad, Lahore Campus, Lahore 54000, Pakistan; 5 Department of Biomedical Sciences, Faculty of Health Sciences, Asia Metropolitan University, 81750 Johor Bahru, Malaysia; 6 Department of Zoology, College of Science, King Saud University, Riyadh 11362, Saudi Arabia; 7 Department of Zoology, Abdul Wali Khan University, Mardan 23200, Pakistan

**Keywords:** C1818T, diabetes, glucose metabolism, G395A, *klotho*

## Abstract

Objective: The objective was to study the association of *Klotho* gene G395A and C1818T single nucleotide polymorphisms with glycemia, serum, glycosylated hemoglobin (HbA1c) level and the risk of type 2 diabetes mellitus (T2DM) in the Pashtun population of Pakistan. Methods: In this study, 195 normal individuals and 217 T2DM patients were enrolled. All subjects were divided into three groups, namely overall subjects (control + T2DM patients), control individuals and T2DM patients, and their fasting glucose, HbA1c level, lipid profile and C1818T and G395A polymorphisms were determined. Results: The allele frequencies of G395A in overall subjects were 0.568 for A and 0.432 for G. Similarly, allele frequencies for G395A in overall subjects were 0.597 and 0.403 for C and T alleles, respectively. The AA genotype of G395A was observed to be a risk factor for T2DM. In normal individuals, no significant (*p* > 0.05) association was observed between klotho C1818T and G395A polymorphisms and hyperglycemia. In overall subjects, the C1818T polymorphism was associated (*p* < 0.05) with high fasting glucose and HbA1c levels in female subjects only. In T2DM patients, both C1818T and G395A polymorphisms were found to be significantly (*p* < 0.05) associated with high fasting glucose and HbA1c levels both in males and females. Conclusion: The G395A polymorphism was observed to increase the risk of T2DM. Both C1818T and G395 were associated with high fasting glucose and HbA1c levels in T2DM patients.

## 1. Introduction

Klotho is one of the important genes located on chromosome 13 and consists of five exons. It encodes a single-pass transmembrane glycoprotein with a molecular weight of 135 kDa [1,2]. The predominant expression of the klotho gene is reported to be in the kidneys and brain. It has also been reported to be expressed in reproductive and endocrine organs [3]. Klotho is an anti-aging protein and plays an important role in increasing life span [4]. It also plays an important role in suppressing various types of cancers [5], regulates calcium and phosphorus homeostasis [6], controls kidney functions and regulates cellular responses [4]. Some previous research also suggests a link between klotho and glucose metabolism [7]. Patients with type 1 diabetes mellitus show decreased klotho levels compared to the normal population [8]. Klotho has also been reported to have a role in regulating the diabetes-associated signaling of intracellular insulin/insulin-like growth factor 1 (IGF 1) [1].

Various studies have been carried out to evaluate the relationship between klotho gene polymorphism and age-related diseases. More than 10 single nucleotide polymorphisms have been reported in the klotho gene [9,10]. Two important polymorphisms in the klotho gene are G395A (rs1207568) and C1818T (rs564481). C1818T is present in exon 4 and is associated with coronary artery disease, bone mineral density, vascular dysfunction, fasting glucose, lipid levels and blood pressure, and hence it serves as a clinically relevant marker [9,11]. G395A is a mutation in the promoter region of klotho [11]. It is associated with coronary artery disease, hypertension and glucose metabolism [12,13]. Both G395A and C1818T polymorphisms in klotho gene in different populations have been reported to be associated with higher fasting glucose levels [7,14].

Diabetes mellitus is a group of disorders having glucose intolerance in common [15]. Currently, about 200 million people are suffering from diabetes, and the number is predicted to increase to 333 million by 2025 [16]. A recent survey reports that 16.98% of the Pakistani population is suffering from type 2 diabetes mellitus (T2DM) while 10.91% are pre-diabetics [17]. In addition, the number of people suffering from hyperglycemia is increasing day by day. Furthermore, the genetic factor that serves as the leading cause of glucose-related disorders in Pakistan is unknown. The role of klotho gene polymorphisms (G395A and C1818T) and their association with serum glucose level and prevalence of T2DM have not been reported in the Pakistani population. This study was thus designed to determine the association of klotho gene G395A and C1818T polymorphisms with serum glucose, HbA1c levels and the prevalence of T2DM in the Pashtun population of Khyber Pakhtunkhwa, Pakistan.

## 2. Materials and Methods

### 2.1. Samples Collection

Overall, 412 subjects (195 normal individuals, including 111 males and 84 females, and 217 type 2 diabetic (T2DM) patients, including 122 males and 95 females) were enrolled in this study. The sample size was calculated using World Health Organization (WHO) formula [18]. Subjects from both genders, age 20–50 years, belonging to Pashtun ethnicity and having documentary evidence of T2DM were included in this study. Subjects from mixed ethnic backgrounds and having comorbidity were excluded. Blood samples were collected in 5 mL EDTA tubes and subjected to various biochemical analyses and then stored at −20 °C for further investigation. A questionnaire was designed for obtaining the data including, age, gender, body mass index (BMI), disease history and family history of the subjects. Informed consent of the subjects was obtained on a predesigned form. The study was approved (No. 314/EC/F.LIFE/UOP-2020) by the ethical committee of the university.

### 2.2. Biochemical Analysis

Serum glucose; HbA1c; and lipid profile including total cholesterol, triglycerides, and high-density lipoprotein were determined using clinical diagnosis kits (Roche Diagnostic, Basel, Switzerland). Blood samples were processed as per manufacturer instructions and then subjected to analysis using the Cobas c311/c501 system (Roche Diagnostics Switzerland). Serum low-density lipoprotein (LDL) was calculated using the following formula:

LDL = TC − (HDL + TG/5)

### 2.3. Analysis of Klotho Gene C1818T and G395A Polymorphisms

DNA was extracted from blood using the phenol–chloroform method and quantified using a UV–Visible spectrophotometer (752 PC, Shanghai, China). Allele-specific primer pairs were used for C1818T and G395A. The variants were amplified by conventional polymerase chain reaction (PCR, MultiGene OptiMax Labnet International, New Jersey, USA) using a DNA amplification kit (Thermo Fisher Scientific, MA, USA). A reaction mixture (20 μL) consisting of 0.25 μL dNTPs, 1.6 μL MgCl_2_, 2 μL buffer, 1 μL of each primer, 0.4 μL Taq polymerase, 12.25 μL water and 1.5 μL DNA was subjected to polymerase chain reaction using allele-specific primers [5]. The PCR-amplified fragments were electrophoresed on 2% agarose gel, stained with ethidium bromide and visualized using a UV-trans illuminator (Wealtec, NV, USA). The CC (291 bp), CT (291 and 179 bp) and TT (179 bp) genotypes of C188T were identified on agarose. The homozygous GG and AA alleles of G395A were identified as 175 and 121 bp fragments, respectively, while the heterozygous GA allele was identified as 175 and 121 bp fragments on agarose gel.

### 2.4. Statistical Analysis

Minitab (R) software was used for statistical analysis. All the biochemical parameters were expressed as mean ± SD. *p* value < 0.05 was considered significant. The differences between demographic and biochemical parameters were analyzed using Student’s *t*-test. Confidence interval (CI) 95% and odd ratios (ORs) were calculated to determine the correlation between various biochemical parameters and klotho gene polymorphism.

## 3. Results

### 3.1. General Characteristics and Biochemical Profiles of Normal Individuals and Diabetic Patients

General characteristics and biochemical profiles of normal individuals and diabetic patients are given in Table 1. The mean age of normal individuals and diabetic patients (43.9 ± 11 and 45.5 ± 6.5 years, respectively) and mean body mass index (BMI) values (24 ± 3.2 and 25.3 ± 3.0, respectively) were not different significantly (*p* > 0.05). Various biochemical parameters, including fasting glucose levels, HbA1c levels and lipid profile (cholesterol, triglycerides, high-density lipoprotein (HDL) and low-density lipoprotein (LDL)), of diabetic patients were significantly higher than those of normal individuals (*p* > 0.05).

### 3.2. Frequency of G395A (rs1207568) and C1818T (rs564481) Polymorphisms in Normal Individuals and Diabetic Patients and Their Association with Risk of T2DM

**Figure 1:**
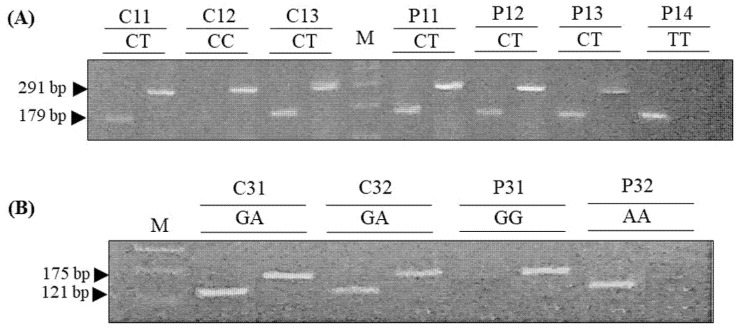
Representative images of klotho C1818T and G395A genotyping. (**A**) The CC genotype of C1818T was identified as a 291 bp fragment, the CT genotype was identified as 291 and 179 bp fragments and the TT genotype was identified as a 179 bp fragment. (**B**) The homozygous GG and AA genotypes of G395A were identified as 175 and 121 bp fragments, respectively, while the heterozygous GA genotype was identified as 175 and 121 bp fragments. Representative image has been shown for control (Subject ID: C11-C13, C31-C33) and T2DM patients (Subject ID: P11-P14, P31, P32). M: Marker (DNA ladder).

The G395A and C1818T single nucleotide polymorphisms were determined both in control and T2DM patients (Figure 1). To evaluate the effect of klotho C1818T and G395A polymorphisms on blood glucose, HbA1c and risk of T2DM, all subjects were divided into three groups: overall subjects (control + diabetics), normal individuals (control) and diabetics (type 2 diabetic patients). The mean fasting glucose and HbA1c levels in wild-type (GG in G395A and CC in C1818T) genotypes were compared with heterozygous mutant (GA in G395A and CT in C1818T) and homozygous mutants (AA in G395A and TT in C1818T). Homozygous mutants (AA and TT) were rare; the homozygous and heterozygous alleles were therefore grouped in each polymorphism (CT+TT and GA+AA) to study the collective effect of the mutant allele of each polymorphism on glucose and HbA1c levels.

The risk association between G395A polymorphism and diabetes is shown in Table 2. Among the overall 195 control subjects, the homozygous wild-type (GG) genotype was present in 77 subjects, the heterozygous mutant genotype (GA) was present in 99 subjects and the homozygous mutant genotype (AA) was present in 19 subjects. Among 217 diabetic patients, GG genotype was present in 28 patients (18 males and 10 females), GA was present in 159 patients (88 males and 71 females) and AA genotype was present in 30 patients. The mutant allele of G395A (A allele carriers) was more prevalent compared to the wild type (GG). Moreover, 75% of overall subjects, 61% of normal individuals and 87% of diabetic patients had the A allele (GA+AA). Allele frequencies for G395A in overall subjects were 0.57 and 0.43 for G and A alleles, respectively. G allele frequencies in the control and diabetic groups were 0.65 and 0.50, respectively while the frequencies of the A allele were 0.35 and 0.50 in the respective groups (Table 3). The presence of mutant genotypes (GA/AA/GA+AA) was associated with higher risk of diabetes in overall subjects (OR = 4.42, *p* < 0.0001, CI = 2.68 to 7.28 for GA; OR = 4.34, *p* = 0.0001, CI = 2.12 to 8.91 for AA; OR = 4.40, *p* < 0.0001, CI = 2.70 to 7.19 for GA+AA), in males (OR = 4.41, *p* < 0.0001, CI = 2.31 to 8.40 for GA; OR = 2.92, *p* = 0.02, CI = 1.19 to 7.19 for AA; OR = 4.09, *p* < 0.0001, CI = 2.19 to 7.65 for GA+AA) and in females (OR = 4.59, *p* = 0.0002, CI = 2.06 to 10.22 for GA; OR = 8.68, *p* = 0.0007, CI = 2.50 to 30.16 for AA; OR = 4.97, *p* = 0.0001, CI = 2.25 to 10.97 for GA+AA).

The risk association between C1818T polymorphism and diabetes is shown in Table 3. Among 195 control subjects, 49 (25%) had the CC genotype and 146 (75%) had the CT genotype. Among 217 diabetic patients, the CC genotype was present in 56 (26%) patients, CT in 136 (63%) patients and TT in 25 (11%) patients. In the overall population (control + diabetics), the mutant T allele (CT+TT) was more prevalent (74%) than the wild-type (CC) genotype (26%). In non-diabetic controls, the homozygous mutant genotype (TT) was not observed in any subject, but this polymorphic form was present in diabetic patients; however, the combined heterozygous CT+TT variant was observed to be 75% prevalent in control and 74% prevalent in diabetic patients. The allele frequencies in overall subjects (control + diabetics) for C1818T were 0.60 for C and 0.40 for T. In the control population and in diabetic individuals, the allele frequencies for C were 0.63 and 0.57, respectively, while allele frequencies for T in control and diabetic patients were 0.37 and 0.43, respectively (Table 2). The mutant genotype (CT/TT) was not associated with higher risk of diabetes in overall subjects (OR = 0.82, *p* = 0.89, CI = 0.52 to 1.28 for CT; OR = 0.97, *p* = 0.88, CI = 0.62 to 1.50 for CT+TT) and in males (OR = 0.47, *p* = 0.02, CI = 0.25 to 0.88 for CT; OR = 0.52, *p* = 0.04, CI = 0.28 to 0.98 for CT+TT). In females, the presence of the heterozygous genotype (CT) was not associated with a higher risk of diabetes (OR = 1.56, *p* = 0.20, CI = 0.79 to 3.06), but when the collective effect of the heterozygous and homozygous mutant (CT+TT) was assessed, the CT+TT was found to be associated with a higher risk of diabetes in female subjects (OR = 1.98, *p* = 0.04, CI = 1.01 to 3.86).

### 3.3. Association of G395A and C1818T Polymorphisms with Serum Glucose and HbA1c Levels

The effect of klotho G395A and C1818T polymorphisms on serum glucose level was evaluated by comparing the blood glucose levels of overall subjects, normal individuals and T2DM patients of various polymorphic forms. Homozygous mutants (AA and TT) were rare; therefore, the dominant genetic models GG vs. GA+AA and CC vs. CT+TT were considered for evaluating the association between C1818T and G395A polymorphisms and serum glucose levels.

The association of G395A and C1818T polymorphisms with fasting glucose and HbA1c levels in overall subjects is shown in Table 4. Among overall subjects, the CC carriers of C1818T SNP had significantly (*p* < 0.0001) lower fasting blood glucose (117.4 ± 48.9 mg/100 mL) and HbA1c (6.0 ± 2.4) levels than individuals carrying the homozygous TT genotype (178.2 ± 37.1 mg/100 mL and 178.2 ± 37.1, respectively), while the heterozygous CT mutation did not show significant impact (*p* > 0.05) on glucose or HbA1c levels. Moreover, in overall female subjects, the TT genotype also showed significantly (*p* < 0.0001) high levels of glucose (215 ± 3.4 mg/100 mL) and HbA1c (11.2 ± 1.4) compared to the wild-type CC genotype (102.9 ± 41.8 mg/100 mL and 4.7 ± 1.5, respectively). Furthermore, the combined CT+TT allele was also in linear correlation (*p* < 0.05) with high blood glucose and HbA1c levels in overall subjects and female individuals, while in male individuals the effect of klotho gene C1818T polymorphism on fasting blood glucose and HbA1c levels was non-significant (*p* > 0.05), which indicates that individuals with the TT genotype are more prone to hyperglycemia and high glycated hemoglobin. In the overall population (control + diabetics), fasting glucose levels in GG, GA, AA and GA+AA allele carriers were 108.2 ± 47.9, 155.8 ± 81.1, 116.3 ± 31.1 and 149.8 ± 76.6 mg/100 mL, respectively, while HbA1c levels were 113.1 ± 51.9, 163.4 ± 82.9, 116.7 ± 29.1 and 154.6 ± 77.9, respectively. The GA, AA and GA+AA genotypes were associated with high blood glucose and HbA1c levels (*p* < 0.05) in overall subjects. Interestingly, in male and female subjects separately, the homozygous AA allele carriers were more prone to hyperglycemia as indicated by significantly (*p* < 0.05) high levels of glucose (153.4 ± 42.9 and 146.4 ± 76.8 mg/100 mL, respectively) and HbA1c (7.5 ± 3.8 and 7.8 ± 4.4, respectively) compared to the GG genotype.

The Association of C1818T and G395A polymorphisms with fasting glucose and HbA1c levels in normal individuals (control) is shown in Table 5. Both the CT and TT genotypes of C1818T and mutant alleles (GA and AA) of the G395A single nucleotide polymorphism were not significantly (*p* > 0.05) associated with fasting blood glucose and HbA1c in the control population, male subjects alone or female subjects alone.

The association of C1818T and G395A polymorphisms with fasting glucose and HbA1c levels in diabetic patients (T2D) is shown in Table 6. The data showed that the CC genotype of klotho in T2DM patients was associated with low fasting glucose (159.3 ± 45.7 mg/100 mL) and HbA1c (8.5 ± 1.8) levels compared to TT (glucose 211 ± 72.9 mg/100 mL and HbA1c 10.2 ± 2.2) and CT (glucose 178.2 ± 37.1 mg/100 mL and HbA1c 9.9 ± 1.6) genotypes. The effect of the heterozygous CT+TT genotype was also significant (*p* < 0.05). The data further showed that the TT genotype was also associated with high blood glucose and HbA1c levels in male and female diabetic patients separately, while CT showed a significant effect in females only. Association analysis of G395A with glucose and HbA1c levels in T2DM patients revealed that patients carrying the AA genotype were more hyperglycemic and also had high levels of HbA1c compared to wild-type GG and heterozygous GA (*p* < 0.05). It is worth mentioning that the combined effect of GA+AA on blood glucose and HbA1c levels was also significant (*p* < 0.05).

## 4. Discussion

Klotho is an anti-aging gene that encodes an aging suppressor protein. The link between klotho and various aging-related pathologies is well established. The C1818T in and G395 single nucleotide polymorphisms have been observed to be associated with a number of physiological functions, including glucose and lipid metabolism [9,11,12,13]. A case–control study was conducted to investigate the frequency of klotho G395A and C1818T polymorphisms and their impact on blood glucose level and the risk of T2DM. In the current study, the allele frequencies of C1818T in overall subjects were 0.5971 for C and 0.4029 for T (Table 2). Similarly, allele frequencies for G395A in overall subjects were 0.568 and 0.432 for G and A alleles, respectively (Table 3). In the Chinese population, the reported allele frequencies for G and A alleles were 0.809 for G and 0.191 for A [13]. In healthy Japanese subjects, the frequency of the A allele was observed to be 0.168, and the frequency of the T allele was 0.177 [14]. Rhee et al. investigated klotho polymorphism in Korean women. The study reported the G and A allele frequencies as 0.829 and 0.171, respectively; in the C1818T polymorphism, frequencies were reported as 0.804 for the C allele and 0.196 for the T allele [7]. Shimyoma et al. investigated klotho polymorphism in Japanese subjects undergoing hemodialysis and reported the frequencies of 0.847 for the G allele and 0.153 for the A allele of G395A and 0.829 for the C allele and 0.171 for the T allele of C1818T [19].

In overall subjects, CT+TT genotypes of C1818T were significantly associated with altered glucose metabolism (high fasting glucose and HbA1c levels) in females, and the GA+AA genotype of G395A was significantly associated with higher mean fasting glucose and HbA1c levels. In T2DM patients, the CT+TT genotype was associated with higher fasting glucose and HbA1c levels, while no significant association between G395A polymorphism and fasting glucose levels or HbA1c levels was observed. G395A polymorphism was found to be associated with a higher risk of T2DM in the overall population as well as in males and females, while C1818T polymorphism was found to be associated with a higher risk of diabetes in female subjects only. The effect of C1818T and G395A polymorphisms on glucose metabolism is also documented in some other populations. Rhee et al. reported that in Korean women, T allele carriers have higher fasting glucose levels [7]. Shimyoma et al. studied the effect of klotho polymorphism on glucose metabolism in healthy Japanese subjects and observed that the T allele of C1818T and the A allele of G395A were associated with high glucose levels in women [14].

The mechanisms by which C1818T affects glucose metabolism and G395A increases the risk of diabetes are not clear. Klotho inhibits IGF-signaling through an unknown mechanism. It is suggested that insulin may induce secreted klotho which then inhibits its signaling by a negative feedback mechanism and hence can prevent prolonged insulin action [20]. Klotho works as a co-receptor for various fibroblast growth factor (FGF) receptors; mutation in klotho may, therefore, alter the functioning of FGF receptors, which are involved in a number of physiological functions. The mutations in FGF-21 are reported to increase carbohydrate consumption and affect fat and protein metabolism [8]. As kidneys are major expression sites for klotho, klotho can also affect glucose metabolism through regulation of gluconeogenesis by affecting the glucose reabsorption and filtration in kidneys. Furthermore, future studies on the functional relationship between klotho and β-klotho can clarify the way in which klotho affects glucose metabolism, as β-klotho shows sequence similarity with klotho, is mainly expressed in the liver and plays a crucial role in glucose metabolism [21]. Electrophoretic mobility shift analysis on human kidney cells has shown that G→A alteration in the promoter region affected the DNA protein binding. This study suggests that G395A polymorphism can play a functional role in various physiological functions that involve klotho [22]. The A allele in G395A can affect the affinity of transcription factors and hence can ultimately affect the expression levels [23]. Although C1818T is an SNP associated with no amino acid substitution, some previous reports suggest that even a silent mutation in an exonic region may lead to alternative transcripts. This may lead to altered protein function or changed protein expression levels [14]. The possible ways in which it can affect the body includes exon skipping and altered RNA stability leading to a variant with altered functions [23]. Future study is required to elucidate how these polymorphisms increase the risk of glucose-related disorders and alter the glucose metabolism in diabetic patients.

The effect of C1818T polymorphism on T2DM was gender-specific, as only female subjects with C1818T were at high risk for T2DM development. This gender-specific action of C1818T polymorphism needs further study. A large population and the study of signaling mechanisms involving wild-type and variant forms of klotho C1818T may uncover its effect on T2DM. Moreover, recent advanced genotyping methods should be used to determine the impact of these polymorphisms on T2DM prevalence and other relevant biochemical parameters.

## 5. Conclusions

Klotho G395A polymorphism was significantly associated with a high risk of T2DM in both males and females, while C181T polymorphism was associated with the risk of type 2 diabetes in female subjects in the Pashtun population of Pakistan. Moreover, klotho G395A and C1818T polymorphisms were also linked with high serum glucose and HbA1c levels in diabetic patients. Klotho G395A and C1818T polymorphisms are thus risk factors for the development of T2DM, elevated glycosylated hemoglobin and hyperglycemia.

## Figures and Tables

**Table 1:** General characteristics and biochemical parameters of control and T2DM patients.

Characteristics	Normal Individuals (n = 195)	T2DM Patients (n = 217)	*p* Value
Age (years)	43.9 ± 11.0	45.5 ± 6.5	0.07
BMI	24.0 ± 3.2	25.3 ± 3.0	<0.07
Glucose (mg/100 mL)	85.1 ± 11.9	198.7 ± 68.3	<0.0001
HbA1c	4.3 ± 0.4	9.6 ± 2.5	<0.0001
Cholesterol (mg/100 mL)	173.3 ± 37.6	192.5 ± 37.0	<0.001
Triglycerides (mg/100 mL)	191.4 ± 72.5	214.2 ± 84.3	0.004
HDL (mg/100 mL)	32.2 ± 8.3	35.6 ± 9.8	0.002
LDL (mg/100 mL)	125.5 ± 26.4	135.5 ± 24.1	0.001

**Table 2:** Frequency of klotho G395A polymorphism and its association with risk of T2DM.

Subjects	Control + Diabetics n = 412 (%)	Control n = 195 (%)	Diabetics n = 217 (%)	OR	*p* Value	95% CI
Overall Subjects (Male + Female)						
GG	105 (25)	77 (39)	28 (13)	Referent		
GA	258 (63)	99 (51)	159 (73)	4.42	<0.0001	2.68–7.28
AA	49 (12)	19 (10)	30 (14)	4.34	0.0001	2.12–8.91
GA+AA	307 (75)	118 (61)	189 (87)	4.40	<0.0001	2.70–7.19
Male						
GG	64 (27)	46 (41)	18 (15)	Referent		
GA	139 (60)	51 (46)	88 (72)	4.41	<0.0001	2.31–8.40
AA	30 (13)	14 (13)	16 (13)	2.92	0.02	1.19–7.19
GA+AA	169 (73)	65 (59)	104 (85)	4.09	<0.0001	2.19–7.65
Female						
GG	41 (23)	31 (37)	10 (11)	Referent		
GA	119 (66)	48 (57)	71 (74)	4.59	0.0002	2.06–10.22
AA	19 (11)	5 (6)	14 (15)	8.68	0.0007	2.50–30.16
GA+AA	138 (77)	53 (63)	85 (89)	4.97	0.0001	2.25–10.97
Allele Frequencies						
G Allele	0.57	0.65	0.50	---	---	---
A Allele	0.43	0.35	0.50	---	---	---

**Table 3:** Frequency of klotho C1818T polymorphism and its association with risk of T2DM.

	Control + Diabetics n = 412 (%)	Control n = 195 (%)	Diabetics n = 217 (%)	OR	*p* Value	95% CI
Overall (Male + Female)						
CC	105 (26)	49 (25)	56 (26)			
CT	282 (68)	146 (75)	136 (63)	0.82	0.89	0.52–1.28
TT	25 (6)	---	25 (11)	---	---	---
Males						
CC	56 (24)	20 (18)	36 (30)	Referent		
CT	168 (72)	91 (82)	77 (63)	0.47	0.02	0.25–0.88
TT	9 (4)	---	9 (7)	---	---	---
Females						
CC	49 (27)	29 (35)	20 (21)	Referent		
CT	114 (64)	55 (65)	59 (62)	1.56	0.20	0.79–3.06
TT	16 (9)	---	16 (17)	---	---	---
Allele Frequencies						
C Allele	0.60	0.63	0.57	---	---	---
T Allele	0.40	0.37	0.43	---	---	---

**Table 4:** Association of klotho C1818T and G395A polymorphisms with fasting glucose levels and HbA1c in overall subjects (control + diabetics).

Subjects	CC	CT	TT	CT+TT	*p* Value CC vs. CT	*p* Value CC vs. TT	*p* Value CC vs. CT+TT
All Subjects (Control + Diabetics)							
Fasting Glucose	117.4 ± 48.9	131.9 ± 76.3	178.2 ± 37.1	134.8 ± 75.3	0.07	<0.0001	0.01
HbA1c	6.0 ± 2.4	6.2 ± 3.1	9.7 ± 1.8	6.5 ± 3.2	0.55	<0.0001	0.04
Male							
Fasting Glucose	131.3 ± 51.3	139.4 ± 79.1	141.7 ± 8.5	139.5 ± 76.9	0.47	0.55	0.46
HbA1c	7.0 ± 2.5	6.5 ± 3.4	8.6 ± 1.2	6.6 ± 3.3	0.31	0.06	0.41
Female							
Fasting Glucose	102.9 ± 41.8	121.8± 71.2	215 ± 3.4	128.6 ± 72.7	0.09	<0.0001	0.02
HbA1c	4.7 ± 1.5	5.3 ± 2.6	11.2 ± 1.4	6.3 ± 2.9	0.06	<0.0001	0.0003
	**GG**	**GA**	**AA**	**GA+AA**	***p* Value** **GG vs. GA**	***p* Value** **GG vs. AA**	***p* Value** **GG vs. GA+AA**
All Subjects (Male + Female)							
Fasting glucose	108.2 ± 47.9	126.3 ± 31.1	155.8 ± 41.1	149.8 ± 76.6	0.028	<0.0001	<0.0001
HbA1c	5.5 ± 2.5	6.8 ± 2.1	7.9 ± 4.1	7.7 ± 3.9	0.002	<0.0001	<0.0001
Male							
Fasting glucose	113.1 ± 51.9	122.7 ± 29.1	153.4 ± 42.9	154.6 ± 77.9	0.32	<0.0001	0.0001
HbA1c	5.6 ± 2.7	6.0 ± 2.0	7.5 ± 3.8	7.7 ± 3.6	0.1	<0.0001	<0.0001
Female							
Fasting glucose	100.7 ± 39.8	115.2 ± 35.7	146.4 ± 76.8	143.1 ± 74.1	0.180	0.0004	0.0006
HbA1c	5.4 ± 2.0	6.5 ± 2.1	7.8 ± 4.4	7.7 ± 4.2	0.06	0.0009	0.0009

**Table 5:** Association of klotho C1818T and G395A polymorphisms with fasting glucose and HbA1c levels in normal individuals.

Subjects	CC	CT	TT	CT+TT	*p* Value CC vs. CT	*p* Value CC vs. TT	*p* Value CC vs. CT+TT
Overall Control (Male + Female)							
Fasting glucose	83.8 ± 10.4	84.9 ± 12.9	---	---	0.59	---	---
HbA1c	4.3 ± 0.4	4.2 ± 0.3	---	---	0.07	---	---
Male							
Fasting glucose	88.7 ± 8.9	88.0 ± 9.8	---	---	0.77	---	---
HbA1c	4.3 ± 0.3	4.3 ± 0.1	---	---	1.00	---	---
Female							
Fasting glucose	81.2 ± 10.2	81.3 ± 14.9	---	---	0.97	---	---
HbA1c	4.2 ± 0.4	4.1 ± 0.3	---	---	0.20	---	---
	**GG**	**GA**	**AA**	**GA+AA**	***p*** **Value** **GG vs. GA**	***p*** **Value** **GG vs. AA**	***p*** **Value** **GG vs. GA+AA**
Overall Control (Male + Female)							
Fasting glucose	85.9 ± 11.1	87.1 ± 9.8	86.7 ± 8.8	87 ± 9.7	0.45	0.77	0.47
HbA1c	4.3 ± 0.4	4.3 ± 0.3	4.5 ± 0.4	4.4 ± 0.4	1.00	0.058	0.09
Male							
Fasting glucose	86.6 ± 11.1	89.6 ± 9.3	89.4 ± 8.3	89.6 ± 9.1	0.15	0.39	0.12
HbA1c	4.3 ± 0.4	4.4 ± 0.3	4.6 ± 0.1	4.4 ± 0.3	0.16	0.8	0.14
Female							
Fasting glucose	85 ± 11.2	84.5 ± 9.6	80.0 ± 6	84.0 ± 9.4	0.83	0.34	0.66
HbA1c	4.3 ± 0.4	4.2 ± 0.4	4.5 ± 0.7	4.3 ± 0.4	0.28	0.36	1.00

**Table 6:** Association of klotho C1818T and G395A polymorphisms with fasting glucose and HbA1c levels in T2DM patients.

Subjects	CC	CT	TT	CT+TT	*p* Value CC vs. CT	*p* Value CC vs. TT	*p* Value CC vs. CT+TT
Overall Diabetics (Male + Female)							
Fasting Glucose	159.3 ± 45.7	178.2 ± 37.1	211 ± 72.9	206.5 ± 69.7	0.03	<0.0001	<0.0001
HbA1c	8.5 ± 1.8	9.9 ± 1.6	10.2 ± 2.2	10.1 ± 2.1	0.001	<0.0001	<0.0001
Male							
Fasting Glucose	156.2 ± 49.5	141.7 ± 8.5	212.9 ± 76.6	204 ± 75.5	0.39	0.0001	0.0007
HbA1c	8.6 ± 1.9	8.9 ± 0.8	810.5 ± 2.7	10.1 ± 2.6	0.65	0.0002	0.002
Female							
Fasting Glucose	168.7 ± 29.6	209.4 ± 66	214.7 ± 3.4	210.5 ± 59.1	0.009	<0.0001	0.003
HbA1c	8.1 ± 0.8	9.6 ±0.7	11.2 ± 1.4	10.0 ± 1.2	<0.0001	<0.0001	<0.0001
	**GG**	**GA**	**AA**	**GA+AA**	***p* Value** **GG vs. GA**	***p* Value** **GG vs. AA**	***p* Value** **GG vs. GA+AA**
Overall Diabetics (Male + Female)							
Fasting Glucose	189.1 ± 40.4	195.9 ± 10.7	211.9 ± 69	202.2 ± 68.1	0.12	<0.0001	0.0040
HbA1c	8.9 ± 0.9	9.2 ± 2.3	10.7 ± 3.5	10.3 ± 3.3	0.06	0.002	0.017
Male							
Fasting Glucose	190.8 ± 44.3	194 ± 11.6	211.7 ± 73.7	200.8 ± 72.1	0.3	0.0002	0.005
HbA1c	8.9 ± 1	9.3 ± 2.6	10.6 ± 2.9	10.2 ± 2.8	0.18	0.001	0.040
Female							
Fasting Glucose	187 ± 28	190.5 ± 5.5	212.1 ± 60.7	205.3 ± 60.5	0.20	0.0001	0.015
HbA1c	8.7 ± 0.4	9.0 ± 0.9	10.7 ± 4.1	10.5 ± 3.9	0.2	0.004	0.023

## Data Availability

All the relevant data are within the manuscript.
